# Is parenthood associated with self-rated health among women in Brazil?

**DOI:** 10.1371/journal.pone.0293262

**Published:** 2023-10-30

**Authors:** Matheus Souza Ferreira, Zilda Pereira da Silva, Marcia Furquim de Almeida, Gizelton Pereira Alencar

**Affiliations:** Department of Epidemiology, University of São Paulo, School of Public Health, São Paulo, Brazil; UNITED STATES

## Abstract

**Background:**

Previous studies conducted in Europe and North America addressing the relationship between self-rated health and parenthood offer inconsistent results, with effects ranging from nonsignificant to significant and in opposite directions. The aim of the present study was to explore the relationship between parenthood and self-rated health (SRH) among women in Brazil (a country with strong inequalities) considering the time interval from the last delivery in the analyses, as proposed in previous studies set in Sweden.

**Methods:**

The study used data from cross-sectional National Health Surveys in Brazil conducted from 2013 to 2014 and 2019 to 2020 with selected groups of 20,046 and 25,100 women for whom complete data were available on the variables of interest. The primary outcome was self-rated health measured on a five-point scale. Partial proportional odds models were employed.

**Results:**

Compared to women that were not a parent, primiparous women whose delivery was within less than one year had a lower likelihood of worse SRH (OR (95% CI): 0.58–0.84 in 2013, and 0.64–0.94 in 2019), whereas multiparous women whose last delivery was more than one year earlier had greater likelihood of worse SRH (OR (95% CI): 1.08–1.27 in 2013, and 1.21–1.39 in 2019).

**Conclusions:**

An association was found between parenthood and SRH among Brazilian women. Considering the epidemiological relevance of SRH, different aspects of parenthood concerning parity and time since the last delivery should be considered in further analyses.

## Introduction

Important changes in lifestyle occur during the transition to becoming a parent. Hypotheses such as the multiple role burden explore the mechanisms by which competing social roles, such as parenthood, marriage, and changes in employment, can affect different aspects of life [1, 2]. Studies investigating the relationship between parenthood and health offer inconsistent findings. Although both significant positive [3–6] and negative [7–9] results have been shown, other studies reported a nonsignificant association [10–13].

Self-rated health (SRH) is a valid health measure that has been extensively used as a universal health indicator as well as a predictor of all-cause mortality [14–19], morbidity status [20], the use of health services [21], and other health outcomes [22–24], as observed in Brazil [25–28]. SRH has been associated with sociodemographic factors, such as household income, age, gender, being married, race/ethnicity, and education level [29–36].

Studies conducted in Sweden [37–39] investigated parenthood and self-rated health, dividing parents into groups based on the time since the last delivery, and highlighted important differences among the groups. Schytt and Hildingsson [37] investigated changes in physical and emotional SRH in late pregnancy, after childbirth, and one year after childbirth in comparison to SRH during mid-pregnancy among women and men in North Middle Sweden. The results showed that women had better SRH after childbirth, but worse SRH after one year in comparison to their SRH during pregnancy. Men had stable SRH after pregnancy and in the first two months of parenthood, followed by worse SRH after one year. The authors suggested possible explanations for such changes in SRH related to motherhood, such as physical and emotional changes, concerns about family finances, returning to work, distress, and having had an emergency cesarean section.

Brazil has had a variety of social, economic, and regional policy strategies concerning family planning, maternal-infant health, and reproductive health [40–42] since the creation of the universal healthcare system in 1990. The country is diverse and, despite the advances that were achieved, considerable regional health inequalities remain [43, 44], along with inequalities in terms of social determinants, especially education, income, and wealth distribution. Such inequalities result in barriers regarding access to health care.

The aim of the present study was to explore the relationship between parenthood and a general measure of SRH in Brazilian women considering the time since the last delivery. Data were used from National Health Surveys (Pesquisa Nacional de Saúde—PNS 2013 and 2019) that covered the health status, lifestyles, and use of services of the population [45, 46], and all the analyses were adjusted by sociodemographic factors.

## Materials and methods

### Data

Publicly available cross-sectional data from the 2013 and 2019 National Health Surveys in Brazil [47, 48] were used in this study. The surveys were conducted from 2013 to 2014 and 2019 to 2020 by the Brazilian Institute of Geography and Statistics and the Health Ministry. Data were collected from respondents of respectively 64,348 (non-response rate: 8.1%) and 108,525 (non-response rate: 6.4%) randomly selected Brazilian households using a complex sampling design [46, 49]. The aims of the survey included the establishment of the health status and lifestyles of the Brazilian population, access to and the use of health services, preventive actions, continuity of care, and health care financing. The questionnaire comprised two modules answered by the residents: the household and socioeconomic situation and general health of all residents; and an individual questionnaire answered by one randomly selected resident aged 18 years or older in 2013 and 15 years or older in 2019 [45, 46, 50].

### Study population

**Fig 1:**
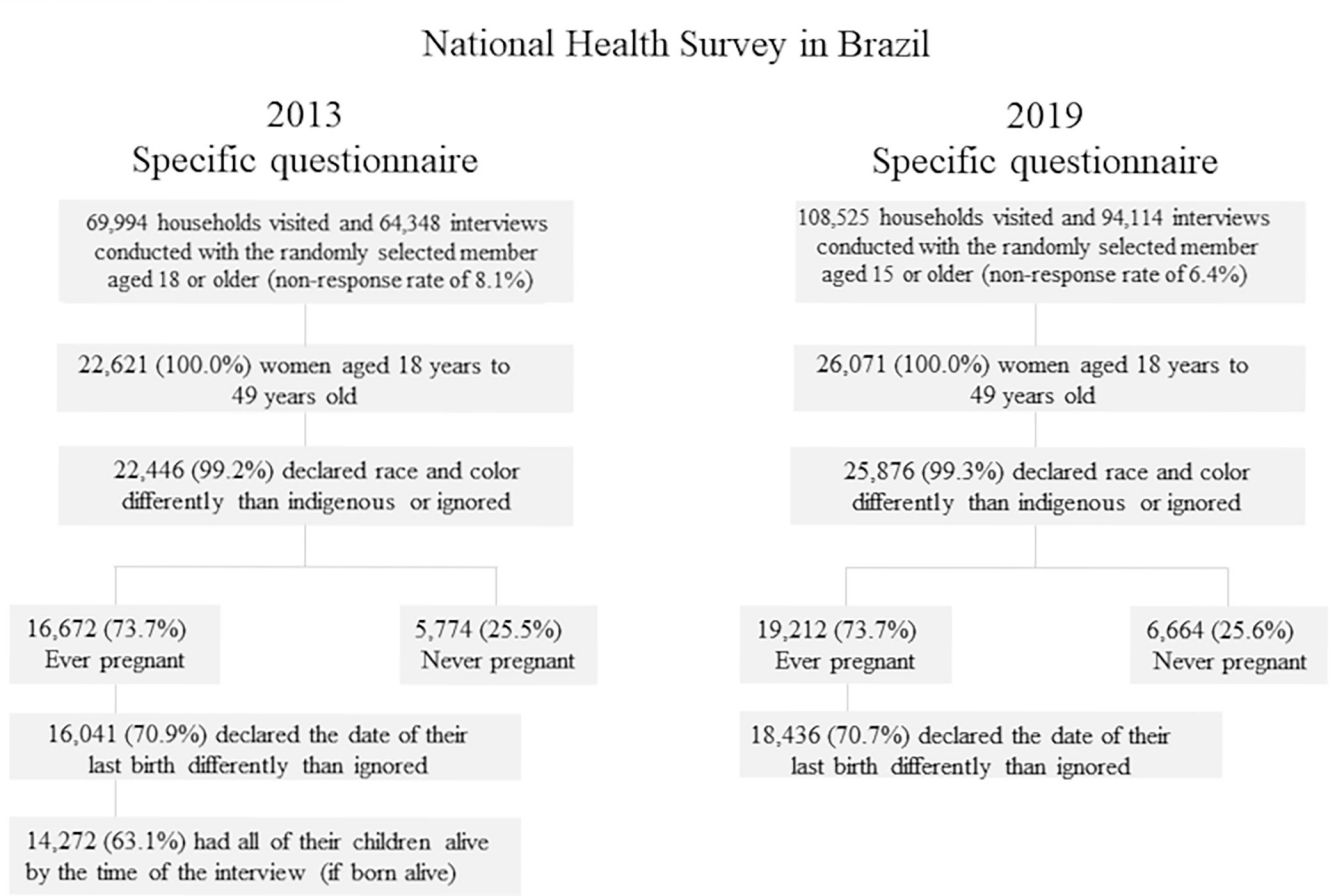
Study population.

For this study, we considered groups of 20,046 women from the 2013 National Health Survey and 25,100 women from the 2019 survey who were up to 49 years old, whose self-declared race/color was other than “unknown” or indigenous (due to dimensioning issues) and who were either never pregnant or, if ever pregnant, did not declare the date of their last birth as “unknown”. Additionally, for the group of women from the 2013 survey, a filter was included of having had all of their children alive, in cases of having had children that were born alive (a possible confounding factor considering the outcome of self-rated health); this variable was not included in the 2019 survey. This process is described in Fig 1.

### Outcome variable

The five-point scale for general self-rated health was the outcome in this study and was included in the two surveys considering the following possible responses: “very good”, “good”, “fair”, “poor”, “very poor”.

### Explanatory variables

Parenthood was measured by combining parity (never pregnant, primiparous, or multiparous) and the time interval between the date of last delivery and when the survey took place, which resulted in a variable with five categories: not a parent; primiparous with delivery less than one year earlier; primiparous with delivery one year earlier or longer; multiparous with last delivery less than one year earlier; and multiparous with last delivery one year earlier or longer.

The control variables considered for the analysis were age (18–24, 25–34, 35–49 years), level of education (no instruction, primary school, high school, university), race/color (White, Black, Yellow, Brown), Brazilian region of residence (Southeast, Northeast, South, North, Midwest), private health insurance (1 year or more, less than 1 year, or none) and living with a partner (yes, no).

### Statistical analyses

Generalized ordered logit includes the partial proportional odds models (PPO) and proportional odds model (PO). These were used to model the ordinal outcome and consider the effect of the explanatory variables over the five-point SRH scale. One usually starts with PO models in which the following (proportional odds) assumption is considered: the coefficients of the binary logistic regressions (“very good” vs. “good”, “fair”, “poor” and “very poor” & “very good” and “good” vs. “fair”, “poor” and “very poor” & “very good”, “good” and “fair” vs. “poor” and “very poor” & “very good”, “good”, “fair” and “poor” vs. “very poor”) all have equal meaning, causing the odds ratios to be the same in all comparisons. If this assumption is not met, the option is to consider PPO models [51]. This assumption was assessed via the Brant test [52] (2013 sample: χ^2^ (21 df.) = 113.56; p-value <0.001; 2019 sample: χ^2^ (21 df.) = 157.30; p-value <0.001). PPO models were estimated with the command “gologit2” using the “autofit” option [53] available in the Stata software version 18.0 [54]. The Nordberg extension of the DuMouchel-Duncan test for generalized linear models [55–57] was used to test if the sampling design was informative for the model and, as suggested, analyses were conducted unweighted (2013 sample: p-value = 0.948; 2019 sample: p-value = 0.595).

### Ethics statement

The National Health Survey 2013 and The National Health Survey 2019 projects were approved by the National Research Ethics Committee/National Health Council (process: 328,159, June 26th, 2013, and process: 3,529,376, August 23rd, 2019). All participants signed the informed consent form.

## Results

### Sociodemographic characteristics

The sociodemographic characteristics of the study population are shown in Table 1. Most of the women were younger than 36 years of age, had completed primary or high school, declared their race/color as Brown or White, lived in the Northeast, Southeast or North regions of Brazil, had no private health insurance at the time of the survey, lived with a partner, were a parent, and self-rated their health as “good” or “fair”.

**Table 1:** Sociodemographic characteristics of selected Brazilian women. 2013 and 2019 National Health Surveys.

Variables	Women—2013		Women—2019		Percentage change (%)
	(n = 20,046)		(n = 25,100)		
	n	%	n	%	
**Age groups (years)**					
18–24	3,997	19.9	4,055	16.2	-18.6
25–35	8,004	39.9	8,951	35.7	-10.5
36–49	8,045	40.2	12,094	48.1	19.7
Total	20,046	100.0	25,100	100.0	
**Level of education**					
University	4,479	22.3	6,778	27.0	21.1
High School	8,327	41.6	10,823	43.1	3.6
Primary school	5,590	27.9	6,905	27.5	-1.4
No instruction	1,650	8.2	594	2.4	-70.7
Total	20,046	100.0	25,100	100.0	
**Race/color**					
White	7,728	38.6	8,296	33.1	-14.2
Black	1,756	8.8	2,825	11.3	28.4
Yellow	192	1.0	182	0.6	-40.0
Brown	10,370	51.6	13,797	55.0	6.6
Total	20,046	100.0	25,100	100.0	
**Region of Brazil**					
Southeast	4,468	22.3	5,012	20.0	-10.3
Northeast	6,143	30.6	8,995	35.8	17.0
South	2,321	11.6	2,859	11.4	-1.7
North	4,572	22.8	5,249	20.9	-8.3
Midwest	2,542	12.7	2,985	11.9	-6.3
Total	20,046	100.0	25,100	100.0	
**Health insurance**					
Longer than 1 year	4,374	21.8	4,666	18.6	-14.7
For 1 year or less	978	4.9	822	3.3	-32.7
No health plan	14,694	73.3	19,612	78.1	6.5
Total	20,046	100.0	25,100	100.0	
**Living with partner**					
Not living with partner	7,924	39.5	10,558	42.1	6.6
Living with partner	12,122	60.5	14,542	57.9	-4.3
Total	20,046	100.0	25,100	100.0	
**Parenthood: parity & time of delivery**					
Not a parent	5,774	28.8	6,664	26.5	-8.0
Primiparous with delivery < 1 year	509	2.5	449	1.8	-28.0
Primiparous with delivery ≥ 1 year	4,352	21.7	5,551	22.1	1.8
Multiparous with last delivery < 1 year	855	4.3	1,048	4.2	-2.3
Multiparous with last delivery ≥ 1 year	8,556	42.7	11,388	45.4	6.3
Total	20,046	100.0	25,100	100.0	
**SRH**					
Very good	2,714	13.5	3,777	15.0	11.1
Good	11,670	58.3	13,411	53.5	-8.2
Fair	4,884	24.4	6,809	27.1	11.1
Poor	651	3.2	889	3.5	9.4
Very poor	127	0.6	214	0.9	50.0
Total	20,046	100.0	25,100	100.0	

In comparison to women from the 2013 sample, the percentage of women in the 36-to-49-year-old age group increased relatively by 19.7%, the group with a university education increased by 21.1%, the group with no instruction decreased by 70.7%, the group that declared their race/color as White decreased by 14.2%, the group that declared their race/color as Black increased by 28.4%, the group that lived in the Northeast region increased by 17.0%, the group with no health insurance increased by 6.5%, and the group that self-rated their health as “good” decreased by 8.2% in the 2019 sample.

### SRH and parenthood

**Fig 2:**
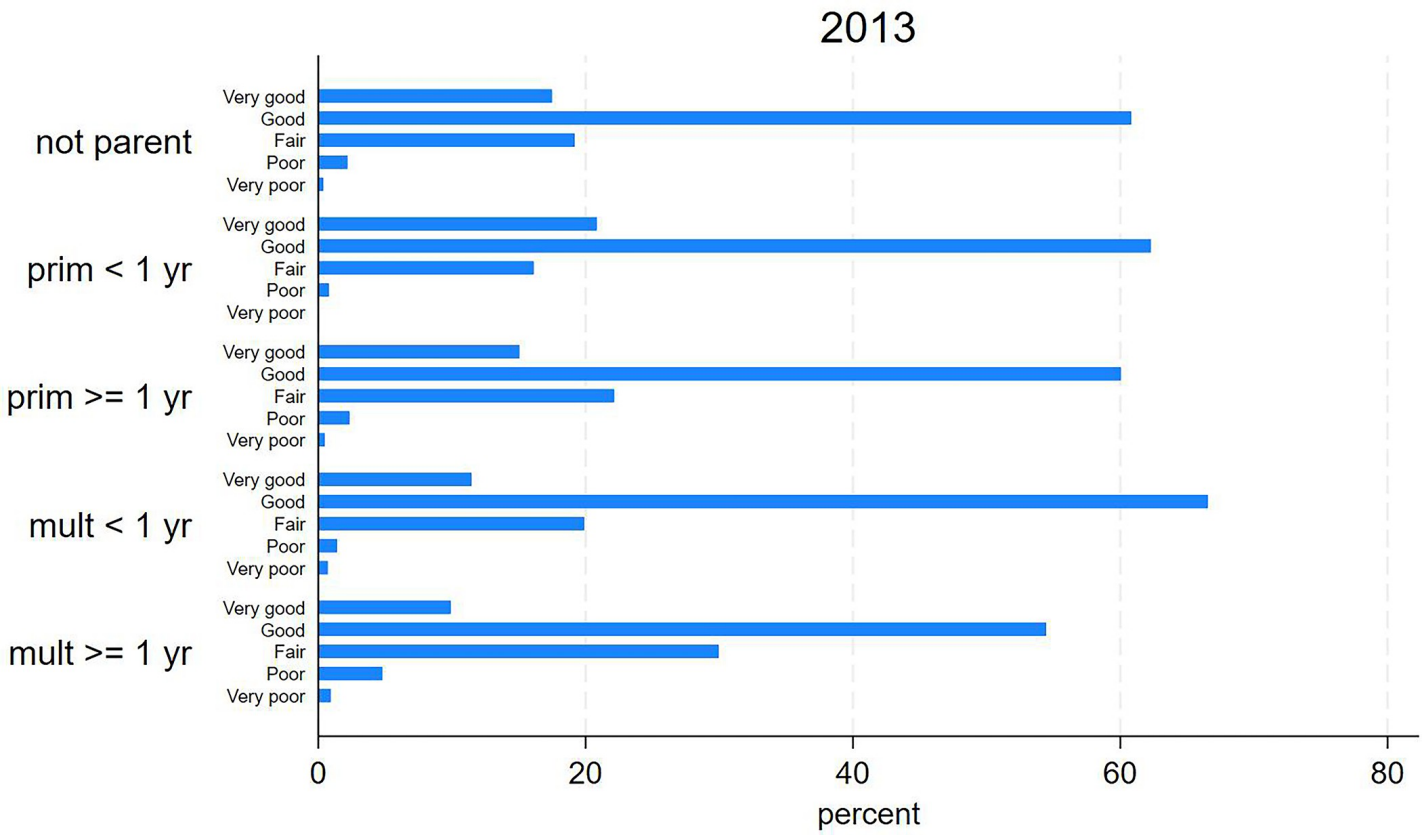
Distribution of selected Brazilian women according to parenthood and self-rated health. National Health Survey, 2013.

**Fig 3:**
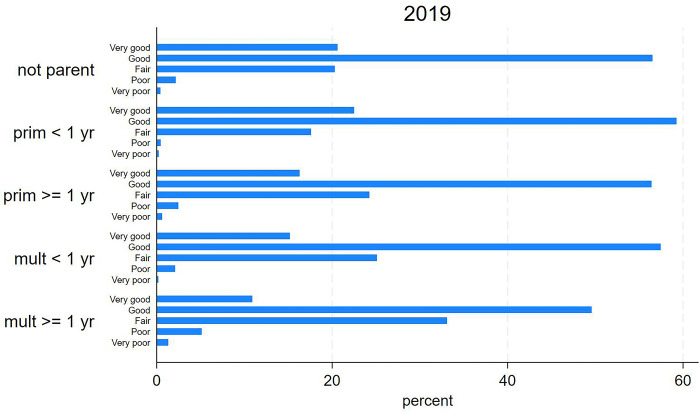
Distribution of selected Brazilian women according to parenthood and self-rated health. National Health Survey, 2019.

Figs 2 and 3 show the distribution of Brazilian women from the 2013 and 2019 health survey samples according to parenthood and SRH. Descriptively, primiparous women had better SRH (higher proportion of “very good/good” and lower proportions of “fair” and “poor/very poor”) than multiparous women. Women with the last delivery within the previous year self-rated their health better than women who were not a parent and those from other groups. Women with the last delivery ≥ one year earlier self-rated their health worse than women who were not a parent and those from other groups. In comparison to the group of women who were not a parent, the highest variations in “poor/very poor” SRH were seen in the group of primiparous women with delivery within the previous year (-78.1% and 2.5% to 0.6%, respectively) and also in the group of multiparous women with last delivery ≥ one year earlier (127.1% and 2.5% to 5.7%, respectively).

### Factors associated with SRH

Tables 2 and 3 display the results of the PPO models for the 2013 and 2019 samples containing the effects expressed as odds ratios (OR) of the explanatory variables on SRH. An older age, lower education levels, “Black” and “Brown” race/color, being a resident of a household in the Northeast, North, and Midwest regions of Brazil, and not having health insurance significantly increased the risk for worse SRH. For the 2013 sample, having health insurance for one year or less also had a significant effect. Living with a partner increased the risk of SRH other than “very good” but also lowered the risk of “very poor” SRH for the 2013 sample and “poor” SRH for the 2019 sample.

**Table 2:** Partial proportional odds models for group of selected women from 2013 National Health Survey sample.

Variables—2013	Self-rated health							
	VG vs G, F, P, VP		VG, G vs F, P, VP		VG, G, F vs P, VP		VG, G, F, P vs VP	
	OR	(95% CI)	OR	(95% CI)	OR	(95% CI)	OR	(95% CI)
**Parenthood: parity & time of delivery**								
Not parent	reference							
Primiparous with delivery < 1 year	0.69**	(0.58–0.84)	0.69**	(0.58–0.84)	0.69**	(0.58–0.84)	0.69**	(0.58–0.84)
Primiparous with delivery ≥ 1 year	0.98	(0.90–1.07)	0.98	(0.90–1.07)	0.98	(0.90–1.07)	0.98	(0.90–1.07)
Multiparous with last delivery < 1 year	1.04	(0.83–1.31)	0.73**	(0.61–0.87)	0.62*	(0.38–1.00)	1.38	(0.60–3.17)
Multiparous with last delivery ≥ 1 year	1.17**	(1.08–1.27)	1.17**	(1.08–1.27)	1.17**	(1.08–1.27)	1.17**	(1.08–1.27)
**Age groups (years)**								
18–24	reference							
25–35	1.11*	(1.02–1.21)	1.11*	(1.02–1.21)	1.11*	(1.02–1.21)	1.11*	(1.02–1.21)
36–49	1.41**	(1.27–1.58)	1.76**	(1.60–1.93)	1.96**	(1.66–2.31)	1.96**	(1.35–2.84)
**Level of education**								
University	reference							
High School	1.54**	(1.42–1.66)	1.54**	(1.42–1.66)	1.54**	(1.42–1.66)	1.54**	(1.42–1.66)
Primary school	2.44**	(2.14–2.77)	2.31**	(2.10–2.55)	3.06**	(2.56–3.66)	3.26**	(2.19–4.85)
No instruction	2.78**	(2.22–3.48)	2.68**	(2.36–3.05)	4.59**	(3.70–5.70)	3.72**	(2.20–6.27)
**Race/color**								
White	reference							
Black	1.20**	(1.08–1.33)	1.20**	(1.08–1.33)	1.20**	(1.08–1.33)	1.20**	(1.08–1.33)
Yellow	1.14	(0.86–1.52)	1.14	(0.86–1.52)	1.14	(0.86–1.52)	1.14	(0.86–1.52)
Brown	1.26**	(1.15–1.38)	1.13**	(1.05–1.21)	0.92	(0.79–1.07)	0.96	(0.67–1.37)
**Region of Brazil**								
Southeast	reference							
Northeast	2.14**	(1.91–2.40)	1.79**	(1.64–1.95)	1.43**	(1.20–1.70)	1.52*	(1.01–2.28)
South	1.05	(0.95–1.16)	1.05	(0.95–1.16)	1.05	(0.95–1.16)	1.05	(0.95–1.16)
North	2.17**	(1.90–2.47)	1.56**	(1.42–1.72)	1.44**	(1.18–1.75)	1.14	(0.70–1.86)
Midwest	1.14**	(1.04–1.26)	1.14**	(1.04–1.26)	1.14**	(1.04–1.26)	1.14**	(1.04–1.26)
**Private health insurance**								
Longer than 1 year	reference							
For 1 year or less	1.31**	(1.14–1.51)	1.31**	(1.14–1.51)	1.31**	(1.14–1.51)	1.31**	(1.14–1.51)
No health plan	1.75**	(1.62–1.90)	1.75**	(1.62–1.90)	1.75**	(1.62–1.90)	1.75**	(1.62–1.90)
**Living with partner**								
Not living with partner	reference							
Living with partner	1.10*	(1.00–1.20)	0.98	(0.92–1.05)	0.94	(0.81–1.09)	0.70*	(0.49–0.99)

*p-value < .05

**p-value < .01

**Table 3:** Partial proportional odds models for group of selected women from 2019 National Health Survey sample.

Variables—2019	Self-rated health							
	VG vs G, F, P, VP		VG, G vs F, P, VP		VG, G, F vs P, VP		VG, G, F, P vs VP	
	OR	(95% CI)	OR	(95% CI)	OR	(95% CI)	OR	(95% CI)
**Parenthood: parity & time of delivery**								
Not parent	reference							
Primiparous with delivery < 1 year	0.78**	(0.64–0.94)	0.78**	(0.64–0.94)	0.78**	(0.64–0.94)	0.78**	(0.64–0.94)
Primiparous with delivery ≥ 1 year	1.07	(1.00–1.15)	1.07	(1.00–1.15)	1.07	(1.00–1.15)	1.07	(1.00–1.15)
Multiparous with last delivery < 1 year	0.93	(0.82–1.07)	0.93	(0.82–1.07)	0.93	(0.82–1.07)	0.93	(0.82–1.07)
Multiparous with last delivery ≥ 1 year	1.30**	(1.21–1.39)	1.30**	(1.21–1.39)	1.30**	(1.21–1.39)	1.30**	(1.21–1.39)
**Age groups (years)**								
18–24	reference							
25–35	1.09*	(1.01–1.18)	1.09*	(1.01–1.18)	1.09*	(1.01–1.18)	1.09*	(1.01–1.18)
36–49	1.30**	(1.18–1.43)	1.61**	(1.49–1.76)	2.19**	(1.89–2.54)	3.25**	(2.32–4.56)
**Level of education**								
University	reference							
High School	1.64**	(1.53–1.75)	1.64**	(1.53–1.75)	1.64**	(1.53–1.75)	1.64**	(1.53–1.75)
Primary school	2.54**	(2.27–2.84)	2.46**	(2.27–2.67)	3.37**	(2.93–3.89)	3.80**	(2.80–5.14)
No instruction	2.69**	(1.92–3.76)	2.81**	(2.36–3.35)	5.31**	(4.10–6.89)	5.53**	(3.21–9.54)
**Race/color**								
White	reference							
Black	1.29**	(1.19–1.41)	1.29**	(1.19–1.41)	1.29**	(1.19–1.41)	1.29**	(1.19–1.41)
Yellow	1.07	(0.81–1.43)	1.07	(0.81–1.43)	1.07	(0.81–1.43)	1.07	(0.81–1.43)
Brown	1.18**	(1.11–1.25)	1.18**	(1.11–1.25)	1.18**	(1.11–1.25)	1.18**	(1.11–1.25)
**Region of Brazil**								
Southeast	reference							
Northeast	1.84**	(1.67–2.02)	1.63**	(1.51–1.76)	1.36**	(1.17–1.57)	1.10	(0.81–1.50)
South	0.86**	(0.78–0.94)	0.86**	(0.78–0.94)	0.86**	(0.78–0.94)	0.86**	(0.78–0.94)
North	1.87**	(1.67–2.09)	1.49**	(1.36–1.62)	1.30**	(1.10–1.55)	0.93	(0.63–1.38)
Midwest	0.97	(0.89–1.06)	0.97	(0.89–1.06)	0.97	(0.89–1.06)	0.97	(0.89–1.06)
**Private health insurance**								
Longer than 1 year	reference							
For 1 year or less	1.15	(0.99–1.33)	1.15	(0.99–1.33)	1.15	(0.99–1.33)	1.15	(0.99–1.33)
No health plan	1.59**	(1.48–1.70)	1.59**	(1.48–1.70)	1.59**	(1.48–1.70)	1.59**	(1.48–1.70)
**Living with partner**								
Not living with partner	reference							
Living with partner	1.10*	(1.02–1.19)	0.97	(0.91–1.03)	0.84**	(0.74–0.95)	0.77	(0.59–1.01)

*p-value < .05

**p-value < .01

In the presence of the other covariates, parenthood had different and statistically significant effects on SRH: being a primiparous woman with delivery less than one year earlier consistently lowered the risk of worse levels of SRH (OR = 0.69 (0.58–0.84) in 2013 and OR = 0.78 (0.64–0.94) in 2019) in comparison to women who were not a parent. Being a multiparous woman with the last delivery one year earlier or longer consistently increased the risk of worse levels of SRH (OR = 1.17 (1.08–1.27) in 2013 and OR = 1.30 (1.21–1.39) in 2019). For the 2013 sample, being a multiparous woman with the last delivery less than one year earlier lowered the risk from “fair, poor and very poor” (OR = 0.73 (0.61–0.87) and “poor and very poor” (OR = 0.62 (0.38–1.00) SRH in comparison to the SRH of women who were not a parent.

**Fig 4:**
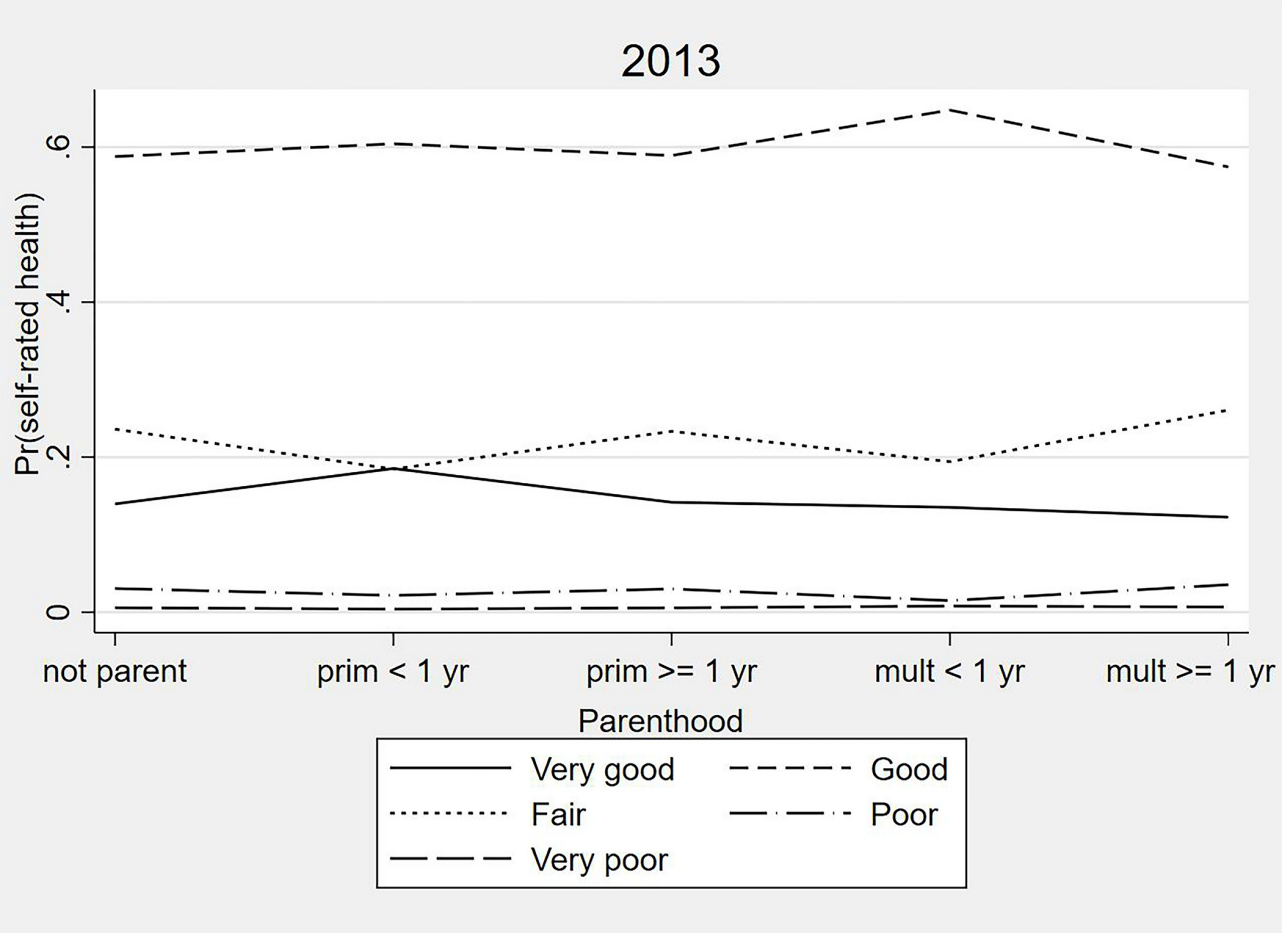
Estimated probabilities of self-rated health according to parenthood from partial proportional odds models for women from the 2013 National Health Survey sample.

**Fig 5:**
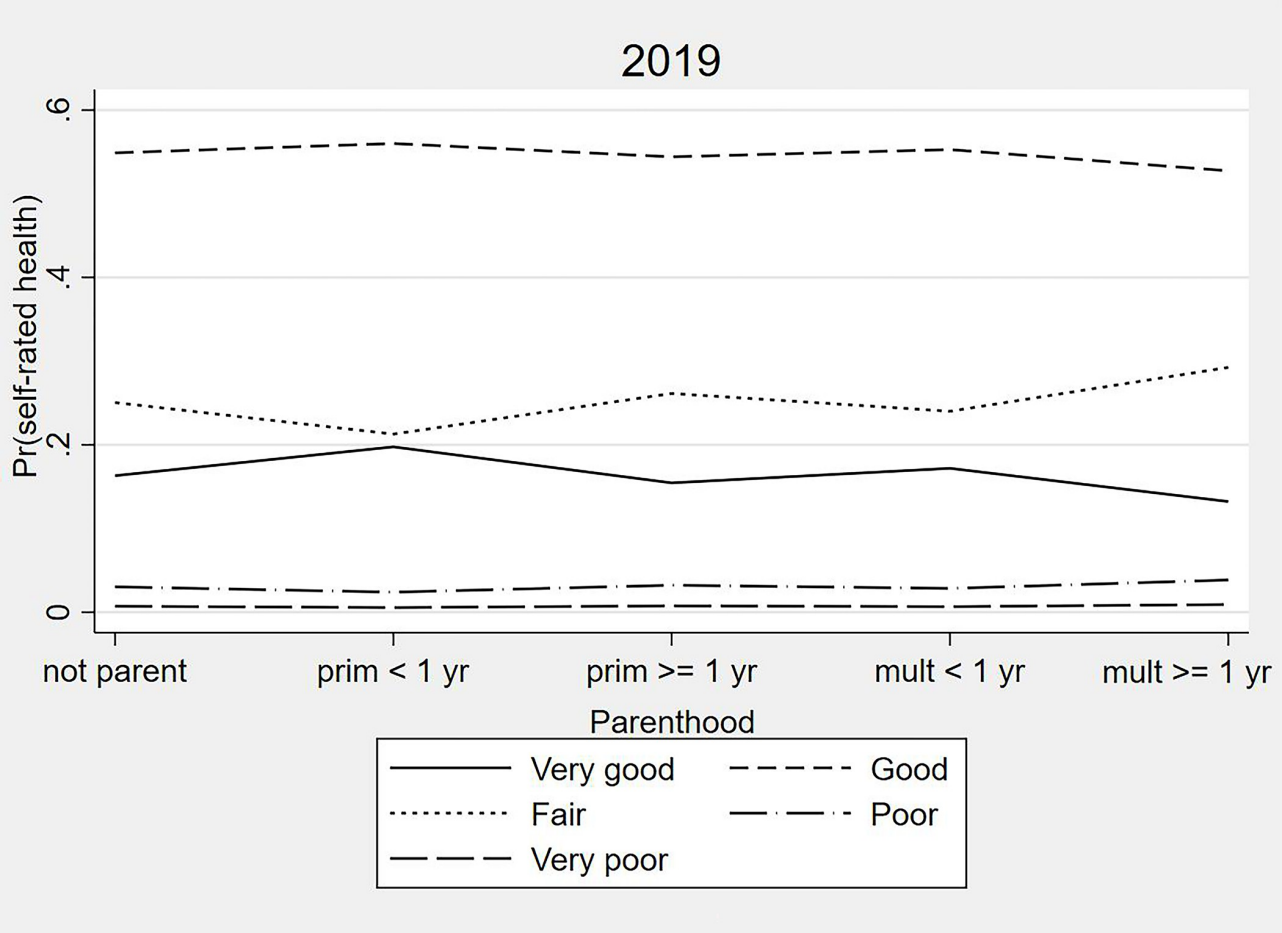
Estimated probabilities of self-rated health according to parenthood from partial proportional odds models for women from the 2019 National Health Survey sample.

Figs 4 and 5 show the model-estimated probabilities of SRH according to parenthood groups from the 2013 and 2019 samples. Primiparous women with delivery ≤ one year earlier were more likely to self-rate their health as “very good” and multiparous women with the last delivery ≤ one year earlier were more likely to self-rate their health as “good” than the other categories. Multiparous women with the last delivery ≥ one year earlier had greater probabilities of “fair”, “poor”, and “very poor” SRH in comparison to other categories of parenthood.

**Fig 6:**
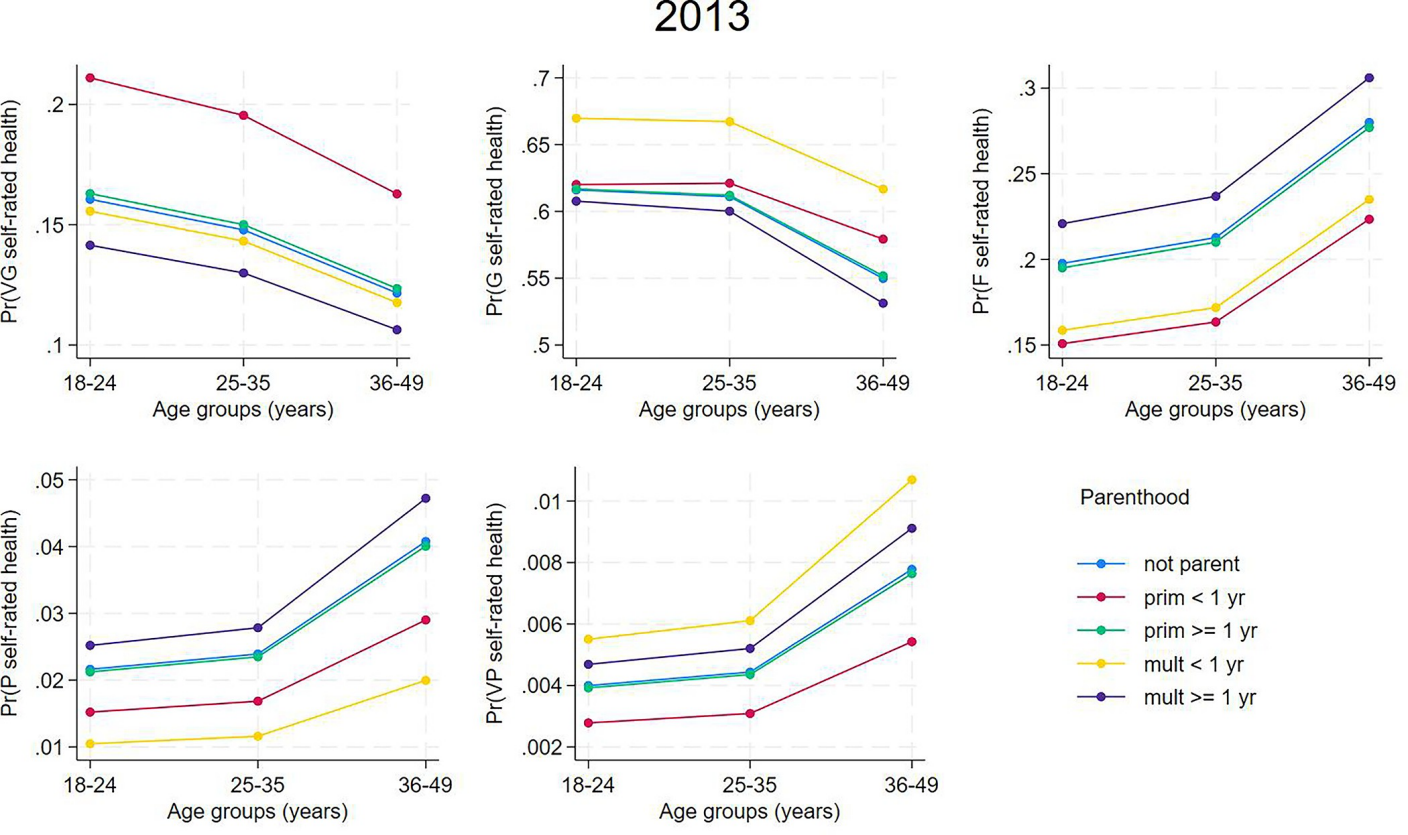
Estimated probabilities of self-rated health according to parenthood and age from partial proportional odds models for women from the 2013 National Health Survey sample.

**Fig 7:**
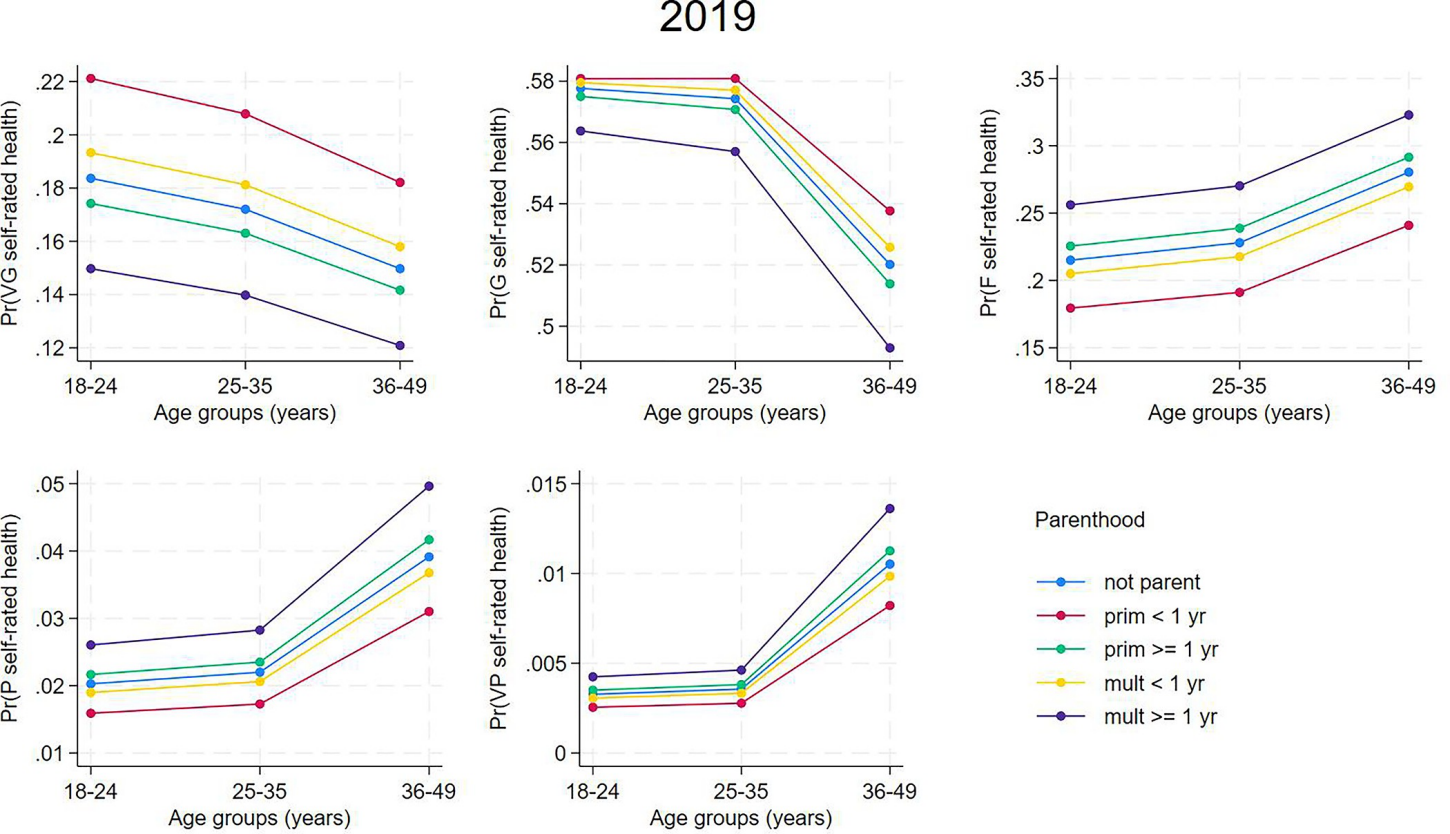
Estimated probabilities of self-rated health according to parenthood and age from partial proportional odds models for women from the 2019 National Health Survey sample.

To visualize the effect of age on the association between SRH and parenthood, we used the predictions from the 2013 and 2019 models to estimate the marginal effects of the variables on the outcome according to age group (18–24, 25–34, 35–49 years) and parenthood (Figs 6 and 7). The findings indicate that age does not affect the association between SRH and parenthood.

## Discussion

Parenthood had a statistically significant effect on the SRH of Brazilian women in both the 2013 and 2019 samples. In comparison to women who were not a parent, having had a delivery within the previous year bettered SRH and this effect was stronger for the group of primiparous women compared to multiparous women. Being a multiparous woman with the last delivery ≥ one year earlier worsened SRH. These significant effects remained in the presence of the sociodemographic control variables age, level of education, race/color, Brazilian region of residence, and having private health insurance.

Studies have shown that social determinants constitute an important factor for several health outcomes and health inequalities have been linked to geography, race/ethnicity, gender, level of education, work situation, socioeconomic status, and other factors [58–61]. Data from the 2013 National Health Survey show that inequalities in education, material deprivation, and the use of healthcare services differed widely among geographic regions of the country, with the worst rates found in the North and Northeast. This inequality was reflected in life-expectancy at birth, which was five years longer in wealthier regions when compared to poorer regions, and rates of poor SRH, which were significantly higher in the latter group [58]. A study [59] using data from 2013 National Health Survey found that the prevalence of unhealthy behaviors was higher among individuals who were non-White, less educated, and had no private health insurance. Another study [60] considering specifically the population of Brazilian mothers (data from 2013 National Health Survey) found that the use of prenatal care differed geographically.

One study [61] showed that, despite an overall improvement in the proportion of the Brazilian population that assessed their SRH as good/very good (53% in 2003 (World Health Survey) versus 66% in 2013 (National Health Survey)), higher rates of poor SRH were found in women compared to men, older individuals compared to younger individuals, non-White individuals compared to White individuals, and individuals with an incomplete primary school education compared to other groups.

The direction of the effects found for the women was similar to the findings of a study conducted in Sweden that investigated the association between parenthood and SRH. It is possible that the similarities in the results of the two studies were due to the classification of different groups of parenthood according to the time since the last delivery and parity.

Studies have mentioned possible mechanisms by which parenthood could affect SRH, primarily among mothers, such as physical and emotional changes [62], changes to self-image, the feeling of being unprepared, multiple role burden, reduction in sexual desirability, the burden of new responsibilities, a lack of support in child-rearing, increased expenses, and concerns and difficulties relating to maintaining jobs, advancing in careers, and achieving professional goals [63].

Studies addressing the association between social roles and health showed that different relationships may be involved concerning the social roles of individuals, especially with regards to the quantity and degree of commitment required. According to the multiple role burden hypothesis, having more social roles would be related to greater burden and stress. In contrast, the role attachment hypothesis states that having more roles would be related to greater engagement in the environment and being more active/healthier; public policies and social welfare systems may also be involved in this relationship.

A study conducted in the USA [64] considering the role burden variables ’job schedule’, ’family dependency’, ’levels of role involvement and responsibility’, etc. indicated that having multiple roles could be associated with better health for both men and women, but being at the ’extremes’ (too low or too high) for explanatory variables could indicate worse health.

A study that considered the populations of Germany, France, and the Netherlands [65] found similar associations between social roles and health in these countries: women who worked part-time in France and the Netherlands had poorer self-rated health than those who worked full-time; men and women living without a partner had poorer self-rated health, as did those living without children at home. Another study considering only the German population [13] found that unemployment was the factor most associated with poorer self-rated health for both men and women. Men with part-time jobs and no children had poorer self-rated health than those who worked full-time. In contrast, no significant difference was found for the group of fathers and groups of women with and without children, although women were more likely to work part-time than men, which indicates that they may participate more in other areas (such as parenthood) and there is a gender difference in the experience of engaging in social roles.

Despite the presence of variables that directly measured the work situation in the 2013 National Health Survey, we opted for using the variable ‘having private health insurance’ as a proxy. Although Brazil has a universal healthcare system, the use of private health services is historically linked to formal employment and individuals with private health insurance were more frequent in the formally employed population compared to the general population (data from 2013 National Health Survey) [66]. The present results showed that the group of women with private health insurance for one year or less and those without insurance were more likely to have SRH other than ‘very good’ compared to those with insurance for more than one year, but no statistically significant effects were found for other categories of SRH.

In our study, the finding that primiparous mothers in their first year of parenthood had a lower risk of poor and very poor SRH and that multiparous mothers with a recent delivery had a lower risk of poor SRH lends support to the role attachment hypothesis (having multiple social roles could be associated with more engagement in the community and better perceived health). On the other hand, multiparous mothers after their first year of parenthood had a greater risk of SRH other than ‘very good’–lending support to the multiple role burden hypothesis, by which accumulating roles, such as going back to work and other activities, would increase burden, making perceived health poorer. In our models, the effects of the control variables on the SRH outcome were strong and statistically significant. This could reflect the prevalent regional, social, and economic inequalities in Brazil.

Another consideration for the direction of the association is that healthier individuals may also have an increased probability of becoming parents [67]. We argue that self-rated health could affect parenthood and that the latter could affect the former, as suggested by the results of the present study considering differences in the levels of SRH between the groups of primiparous and multiparous women within one year and after one year of becoming a parent.

This study was possible due to publicly available data from National Health Surveys, which made the inferential analyses feasible. The use of all five categories of the outcome variable of general SRH in the analyses enabled finding significant effects that would be diluted by combining categories. Given the nature of the outcome, the ordinal models employed in the analyses were a more suitable method for identifying these effects.

### Limitations

This study employed data from a cross-sectional study, which implies analytical and interpretative limitations in comparison to longitudinal designs. In addition, there was no information or only partial information available on other variables, such as the type of delivery that had the “cesarean section” category separated into “planned” and “emergency”, which could have been an important variable in the modeling, considering the long-term consequences of an emergency cesarean on SRH described in a previous study [38].

## Conclusions

Brazilian mothers had better self-rated health in the first year of parenthood compared to women who were not a parent, but worse self-rated health after one year. Primiparous women had better self-rated health compared to multiparous women. These relationships are also related to socioeconomic conditions and the strong iniquities in Brazil were reflected in stronger effects of the control variables than those found in previous studies.

The consistency of these results in comparison to those of previous studies performed in very different socioeconomic settings confirms that the time since the last delivery is a variable that clarifies the relationship between parenthood and self-rated health.
